# Using a decision support tool to reduce inappropriate antibiotic use after a delayed prescription

**DOI:** 10.1111/aphw.70198

**Published:** 2026-08-07

**Authors:** Elisabeth D. C. Sievert, Rian Gross, Lars Korn, Cornelia Betsch, Robert Böhm

**Affiliations:** ^1^ Health Communication, Institute for Planetary Health Behaviour University of Erfurt Erfurt Germany; ^2^ Health Communication, Implementation Research Bernhard Nocht Institute for Tropical Medicine Hamburg Germany; ^3^ Faculty of Psychology University of Vienna Vienna Austria; ^4^ Department of Banking and Finance University of Innsbruck Innsbruck Austria

**Keywords:** antimicrobial resistance, antibiotic stewardship, decision support tool, delayed prescribing

## Abstract

Antimicrobial resistance is partly driven by overprescribing antibiotics in primary care. Delayed antibiotic prescriptions, which allow patients to monitor their symptoms before deciding to use antibiotics, are a promising intervention. However, this strategy is undermined when patients initiate antibiotic use immediately. Using a behavioural game measure with behaviour‐contingent financial incentives that models delayed prescriptions, we examined whether a decision support leaflet issued by the World Health Organization can, alone or in combination with other evidence‐based interventions, reduce inappropriate antibiotic use for viral infections. In a pre‐registered online experiment (*N* = 663 UK adults), participants were randomly assigned to one of five experimental conditions: (i) control condition, (ii) leaflet only, (iii) leaflet with value integration task, (iv) leaflet with active symptom monitoring and (v) leaflet with value integration and active monitoring. Inappropriate antibiotic use was lower in all decision support conditions than in the control condition (*p* < .001, *η*_*G*_^2^ = .02). Decision support tools also reduced decisional conflict (*p* < .001, *η*_*G*_^2^ = .02), particularly by improving value clarity and perceived decisional support. Integrating decision support tools into delayed prescription procedures can contribute to a more informed and responsible approach to antibiotic use by providing relevant information and tools.

## INTRODUCTION

Antimicrobial resistance (AMR) poses a major threat to global health, and it is estimated that by 2050, approximately two million people could die from drug‐resistant infections globally per year (Naghavi et al., 2024). Overprescription and inappropriate use of antibiotics are major contributors to the global increase in AMR (Cassini et al., 2019; Hofer, 2019). Appropriate prescribing decisions are typically defined as the right drug administered at the right time, in the right dose, for the right length of time (Dryden et al., 2011). Hence, prescribing antibiotics is considered inappropriate when, per established guidelines, the use of other or no antimicrobial agents is recommended. For example, prescribing antibiotics to treat viral infections is considered inappropriate (Sijbom et al., 2023).

Delayed antibiotic prescribing is a recommended strategy to reduce inappropriate antibiotic use, particularly in cases where it is uncertain whether the infection is viral or bacterial (Spurling et al., 2017). By encouraging watchful waiting, that is, instructing patients to wait and monitor their symptoms before initiating antibiotic treatment, delayed prescribing has been demonstrated to reduce antibiotic use in primary care settings (Little et al., 2014; Spurling et al., 2017; Stuart et al., 2021). Therefore, it offers an effective compromise between immediate prescribing and withholding treatment altogether. Moreover, delayed prescribing can lead to a shorter symptom duration than withholding antibiotics (de la Poza Abad et al., 2016) and can foster trust in the doctor–patient relationship, because it empowers patients to actively participate in treatment decisions (Dallas et al., 2020).

However, the effectiveness of delayed prescribing is often undermined by low patient adherence: many patients fill and use the antibiotic prescription immediately, even when advised to delay treatment. A systematic review found that nearly two thirds of patients initiated antibiotic use on the same day they received a delayed prescription (Francis et al., 2012), and recent observational data report similarly high rates of immediate use (Llor et al., 2022). This immediate use undermines the intention of delayed prescribing and can lead to unnecessary antibiotic consumption, exposing patients to potential side effects without clinical benefit (Rowe & Linder, 2020). Additionally, unnecessary antibiotic use increases AMR. Hence, there is an imbalance between short‐term individual gain and long‐term collective public health outcomes in the delayed prescribing approach, whereby patients may interpret the act of receiving a prescription as a cue for immediate use. To mitigate this, delayed prescribing should be embedded within a safety netting framework (Jones et al., 2019). This includes clear communication about clinical uncertainty, explicit instructions about when to initiate antibiotics and guidance on when to seek follow‐up care. Therefore, to support more appropriate antibiotic use and increase the effectiveness of delayed prescribing for antibiotic stewardship, complementary communication interventions are necessary to help patients better understand the rationale behind delayed prescribing and empower them to make informed decisions about if and when to start antibiotic treatment. Understanding why patients often fail to adhere to watchful waiting reveals barriers that these communication interventions must address.

There are at least two interrelated explanations for the suboptimal effectiveness of delayed antibiotic prescriptions (Santana et al., 2024): First, patients experience uncertainty regarding the interpretation of their symptoms and which implications this has for the decision to start the antibiotic treatment during the delayed prescription period. Second, people have a need for action, letting them prefer active solutions over inactive approaches. This is referred to as the action bias, which has been shown to contribute to the use of antibiotics under uncertainty (Santana et al., 2024; Thorpe et al., 2020). A previous study using a hypothetical delayed prescription task (Santana et al., 2024) incentivised with financial rewards for delaying antibiotic treatment showed that action bias could be reduced: By actively monitoring their symptoms using a symptom diary, the participants felt less uncertain, and the monitoring activity fulfilled their need for action. Thus, the intervention improved the effectiveness of delayed prescriptions as it led to reduced antibiotic use (Santana et al., 2024).

Decision support tools represent a potential tool for incorporating symptom diaries and similar interventions to address the problems resulting from experiencing uncertainty, action bias and potentially other psychological effects. Decision support tools are designed to help patients make informed health and treatment decisions by providing clear, evidence‐based information and supporting them in understanding their condition and treatment options (Stacey et al., 2024). Thus, decision support tools can help to clarify patients' values, foster their active participation in the decision‐making process (Park et al., 2024) and reduce decisional conflict when having to decide between different treatment options (Coronado‐Vázquez et al., 2020; van Dijk et al., 2021). The process of clarifying and expressing values through value elicitation methods is an important aspect of joint decision‐making and helps people evaluate the desirability of options in a given context (Witteman et al., 2021). This process is therefore a recommended part of patient decision support tools (Witteman et al., 2021). Similarly, decision support tools are intended to promote a more active role for patients in their health‐related decisions, with practices such as keeping a symptom diary (Andrews et al., 2021; Rofail et al., 2022; Santana et al., 2024) encouraging active symptom monitoring.

From a theoretical perspective, the use of decision support tools in the context of delayed antibiotic prescribing can be situated within the COM‐B model of behaviour change (Michie et al., 2011). In this framework, the decision support tool is primarily designed to enhance patients' psychological capability by improving their understanding of their infection and to make decisions under uncertainty about whether antibiotics are needed. By clarifying typical illness trajectories (Eley et al., 2020) and highlighting limited benefits and potential harms for likely viral infections, the leaflet aims to reduce antibiotic expectations driven by uncertainty (Santana et al., 2024; Sievert et al., 2024). At the same time, it offers physical opportunity through a tangible resource (the leaflet) and may strengthen social opportunity if perceived as endorsed by a healthcare professional. Furthermore, it targets reflective motivation by understanding the risks and benefits of antibiotic use and automatic motivation by increasing confidence in delaying antibiotics. This ultimately supports the desired behaviour of reducing inappropriate antibiotic use. In addition, relating to self‐determination theory (Ryan & Deci, 2000), the intervention can promote patients' sense of autonomy by enabling value‐congruent decisions, competence by clarifying which symptoms to look out for and relatedness when the tool is framed as coming from a trusted healthcare professional. All these aspects are critical for sustaining health behaviour change.

Here, we investigate whether using a decision support leaflet can be a useful tool to reduce patients' intentions of inappropriately using antibiotics after receiving a delayed prescription. Specifically, the present study examines whether the use of an existing decision support leaflet issued by the World Health Organization (WHO), and further extensions thereof, can reduce the inappropriate use of antibiotics for a viral infection. To this end, we assess participants' antibiotic use in a behavioural game (Interactive Resistance Game; Santana et al., 2024), that is, an experimental setting that models delayed prescriptions in a hypothetical decision task. The behavioural game measure has the advantage of assessing individual behaviour in a highly controlled setting. Despite the hypothetical nature of the task, behaviour‐contingent financial incentives simulate the decisional conflict that patients face in everyday life decisions about antibiotic use (e.g. Thielmann et al., 2021).

## METHOD

The study's materials, analysis code and data are available on the Open Science Framework (https://osf.io/5hscn). The study with its hypotheses was pre‐registered (https://aspredicted.org/7wdv-nrdr.pdf). Ethical approval was granted by the Department of Psychology at the University of Vienna (2023/W/015A).

### Experimental conditions

All participants played a delayed prescription game in which they must decide across several rounds whether they take an antibiotic or whether they manage ongoing symptoms without medication, in line with the watchful waiting approach. The task is described in more detail below. We implemented a one‐factorial between‐participants design implementing five experimental conditions with random assignment (using SoSci Survey's built‐in randomiser).

Participants in the *control condition* performed the delayed prescription task and received no additional information. Participants in all decision support conditions, in contrast, received an adapted version of the WHO decision support leaflet (https://who.canto.global/v/UFPS6IVVV4/allfiles?viewIndex=0&auth=sso) prior to engaging in the delayed prescription task. The decision support leaflet is based on a previous decision support tool (Ashiru‐Oredope et al., 2020) and is intended to be distributed during general practitioner consultations. It included key elements for patient support tools (Sepucha et al., 2013) such as general information about antibiotics and AMR, a list of present symptoms, the diagnosis given at the doctor's consultation, the doctor's recommendation regarding an antibiotic, recommendations of other (non‐antibiotic) interventions and warning signs of what the patients should look out for and contact information in case symptoms get worse. In our adapted version used in this study, the leaflet was tailored to fit the hypothetical scenario of an ear infection, with symptoms corresponding to those in the behavioural task. We omitted contact details, as this was not applicable for the experimental online setting. The study included four intervention conditions in addition to the control group.

In the *leaflet‐only condition*, participants received the standard decision support leaflet, which was displayed after each decision round to simulate real‐world access to information during the watchful waiting period (Figure S1). This served as the baseline decision support tool condition to evaluate the effectiveness of the leaflet in its original, informational form.

To assess whether tailoring the decision support tool with additional psychologically informed components could further enhance its impact, three additional conditions were developed. In the *value integration condition*, participants were prompted to reflect on their values related to antibiotic use by rating four statements (e.g. ‘I want to explore alternative treatment options before considering the use of antibiotics’, Figure S2). This manipulation draws on literature suggesting that value clarification can enhance decision quality and reduce decisional conflict (Park et al., 2024; Witteman et al., 2021). Moreover, incorporating value clarification aligns with the international standards for high‐quality decision support tools, such as those outlined by the International Patient Decision Aids Standards (IDPAS) Collaboration (Elwyn, 2006).

The *active monitoring condition* incorporated a symptom diary in addition to the leaflet (Figure S3). Symptom tracking has been shown to reduce uncertainty and fulfil patients' need for action, particularly under diagnostic ambiguity (Santana et al., 2024). By recording symptom intensity in the morning and evening, this condition was designed to reduce action bias—a tendency to prefer treatment even when inaction may be more appropriate (Tarrant & Krockow, 2022; Thorpe et al., 2020).

Finally, in the *value integration and active monitoring condition*, participants received both extensions—value elicitation and symptom tracking—alongside the leaflet. This condition tested whether combining both components would have a synergistic effect.

### Hypotheses

We pre‐registered four main hypotheses.

- *Decision support hypothesis*: Participants in the decision support conditions would show less inappropriate antibiotic use than those in the control condition.
- *Monitoring hypothesis*: Participants in the active monitoring condition would use fewer antibiotics than those in the leaflet‐only condition.
- *Value hypothesis*: Participants in the value integration condition would use fewer antibiotics than those in the leaflet‐only condition.
- *Combination hypothesis*: Participants in the value integration and active monitoring condition would use fewer antibiotics than participants receiving only one of the components.

We further explored how receiving the different interventions influenced participants' perceived decisional conflict, a major evaluative construct in the decision support literature (Sepucha et al., 2013).

### Measures

#### Behavioural game

**FIGURE 1:**
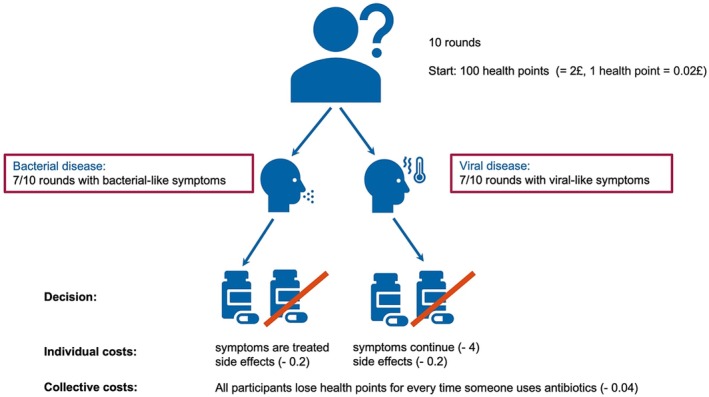
Overview of the delayed prescription task. *Note*: Game‐theoretical equilibrium analysis indicates that antibiotic use is selfish‐rational (but collectively inefficient) in cases of bacterial infection, whereas it is irrational and inefficient in cases of viral infection. Only participants in the viral disease condition were analysed in the present study.

As the central outcome measure, we used the delayed prescription task depicted in Figure 1 (adapted from Santana et al., 2024). In this game, participants suffer from an ear infection for which the doctor gives them a delayed prescription and instructs them to wait and monitor their symptoms before taking antibiotics.

The game involves *n* participants, each endowed with 100 health points (converted into money after the study, conversion rate: 100 points = £2). All participants of the game experience a hypothetical disease, which may be either bacterial or viral. However, the participants were unaware of the nature of their infection; thus, they experienced diagnostic uncertainty regarding the disease group to which they belonged. Throughout 10 rounds, representing the mornings and evenings of five consecutive days, they received information about their symptoms, which were either associated with a viral (‘sore throat’ and ‘muffled hearing’) or bacterial infection (‘severe ear pain’ and ‘purulent fluid from the ear’). In the instructions (which they were able to download before the game), they received an explanation of which symptoms were indicative of which infection. In each condition, the predominant symptoms occurring in 7 out of 10 rounds corresponded to the respective condition's disease type, that is, were indicative of either a bacterial or viral disease; the remaining 3 out of 10 symptoms corresponded to the other disease type. In each round, participants could decide whether to start antibiotic treatment. Crucially, once antibiotic use was initiated, the medication continued to be taken until the end of the game to simulate a full course of antibiotics. This means that a decision to start antibiotics in Round 6, for example, resulted in antibiotic use from Rounds 6 to 10.

The decision affected participants' health points and thus their monetary payoff. Depending on their type of disease and the treatment decision, points were deducted from the initial endowment. That is, participants lost four points in each round with an untreated disease. In the bacterial condition, taking antibiotics prevented further point loss, while in the viral condition, antibiotics were ineffective because they are unable to cure viral infections. Regardless of the condition, antibiotic use led to individual costs due to side effects (0.20 points per round) and collective costs representing AMR (0.04 points per individual using the antibiotic in each round). Optimal individual strategies differ by disease condition: In the bacterial condition, participants should use antibiotics as early as possible. In the viral condition, in contrast, participants should avoid them. Due to the negative social impact of AMR, however, the collectively optimal strategy is to refrain from starting the antibiotic treatment across both disease conditions (further details on the game‐theoretical analysis: Santana et al., 2024).

Because we were mainly interested in inappropriate antibiotic use, we adapted the game so that all participants were assigned to the viral condition of the delayed prescription task, in which any antibiotic intake is inappropriate. To maintain diagnostic uncertainty, participants still had to figure out throughout the game which disease condition they were assigned to. To accurately calculate the bonus payment and avoid deception (Hilbig et al., 2022), we recruited 50 additional participants for the bacterial disease condition whose responses were matched to the study participants. These individuals were not included in the data analysis reported in this paper.

Consequently, the main dependent variable was inappropriate antibiotic use when facing a viral infection, operationalised as the number of rounds during which antibiotics were taken. The decision to start antibiotics was irreversible within the task. Once participants chose to begin treatment, antibiotics were taken in every remaining round. Therefore, the timing of starting antibiotics determined the cumulative score. For instance, starting antibiotic treatment in Round 6 resulted in a score of 5 (i.e. Rounds 6 to 10). Thus, the longer they delayed antibiotic intake in the task, the less antibiotics were used inappropriately because participants took antibiotics in fewer rounds.

#### Decisional conflict

We assessed decisional conflict as an exploratory measure using the 16 items of the Decisional Conflict Scale (O'Connor, 1995). The items include statements such as ‘I know the risks and side effects of each option’ and ‘I feel I have made an informed choice’. The participants rated the items on a scale ranging from 1 (*strongly disagree*) to 5 (*strongly agree*). We calculated separate mean scores for the five subscales feeling informed (Cronbach's *α* = .90), value clarity (*α* = .90), decisional support (*α* = .77), decisional uncertainty (*α* = .88) and effective decision‐making (*α* = .88), with higher scores indicating higher levels of the respective aspects. For the total mean decisional conflict score, we recoded the items so that higher scores indicated greater decisional conflict (*α* = .94).

### Participants

Based on an a priori power analysis reported in the pre‐registration, we recruited a sample of 720 participants from the UK general population via Prolific (https://www.prolific.com/). We excluded participants whose survey responses were unusually fast (*n* = 18) or slow (*n* = 18; lower and upper 2.5% in completion time, respectively). Although we had pre‐registered that participants failing the comprehension check would be excluded, no participants met this criterion. The final sample consisted of *N* = 663 individuals included in the analyses (*n* = 128 in the control condition, *n* = 136 leaflet‐only condition, *n* = 134 value integration condition, *n* = 133 active monitoring condition and *n* = 132 value integration and active monitoring condition).

A comprehensive overview of the participants' characteristics by experimental condition is displayed in Table 1. The participants were aged 19–81 years (*M* = 40 years, *SD* = 12). Among them, 50.2% were female, 48.7% were male and 1.2% identified as non‐binary or other. In terms of education, 62.2% had a bachelor's degree or higher. In terms of occupations, 8.6% stated that they worked in the healthcare sector.

**TABLE 1:** Overview of participant characteristics by treatment condition.

	Control (*n* = 128)	Leaflet only (*n* = 136)	Value integration (*n* = 134)	Active monitoring (*n* = 133)	Value integration and active monitoring (*n* = 132)	Overall (*N* = 663)
Age						
Mean (SD)	40.1 (12.2)	41.8 (13.5)	40.1 (13.1)	40.4 (12.2)	39.7 (12.7)	40.4 (12.7)
Median [min, max]	37.0 [19.0, 68.0]	40.0 [19.0, 81.0]	37.5 [21.0, 80.0]	39.0 [20.0, 72.0]	37.0 [21.0, 81.0]	38.0 [19.0, 81.0]
Gender						
Female	67 (52.3%)	64 (47.1%)	59 (44.0%)	82 (61.7%)	61 (46.2%)	333 (50.2%)
Male	59 (46.1%)	69 (50.7%)	75 (56.0%)	51 (38.3%)	69 (52.3%)	323 (48.7%)
Non‐binary	1 (0.8%)	2 (1.5%)	0 (0%)	0 (0%)	0 (0%)	3 (0.5%)
Prefer to self‐describe	0 (0%)	1 (0.7%)	0 (0%)	0 (0%)	0 (0%)	1 (0.2%)
Prefer not to say	1 (0.8%)	0 (0%)	0 (0%)	0 (0%)	2 (1.5%)	3 (0.5%)
Education						
No schooling completed	0 (0%)	0 (0%)	0 (0%)	0 (0%)	0 (0%)	0 (0%)
Nursery school to 8th grade	0 (0%)	0 (0%)	0 (0%)	0 (0%)	1 (0.8%)	1 (0.2%)
Some high school, no diploma	5 (3.9%)	3 (2.2%)	3 (2.2%)	4 (3.0%)	5 (3.8%)	20 (3.0%)
High school graduate	19 (14.8%)	16 (11.8%)	27 (20.1%)	11 (8.3%)	15 (11.4%)	88 (13.3%)
Some college credit without degree	15 (11.7%)	16 (11.8%)	9 (6.7%)	12 (9.0%)	15 (11.4%)	67 (10.1%)
Trade/technical/vocational training	14 (10.9%)	15 (11.0%)	11 (8.2%)	10 (7.5%)	11 (8.3%)	61 (9.2%)
Associate degree	1 (0.8%)	2 (1.5%)	1 (0.7%)	5 (3.8%)	2 (1.5%)	11 (1.7%)
Bachelor's degree	44 (34.4%)	51 (37.5%)	53 (39.6%)	55 (41.4%)	54 (40.9%)	257 (38.8%)
Master's degree	20 (15.6%)	25 (18.4%)	20 (14.9%)	28 (21.1%)	18 (13.6%)	111 (16.7%)
Professional degree	6 (4.7%)	3 (2.2%)	7 (5.2%)	2 (1.5%)	3 (2.3%)	21 (3.2%)
Doctorate degree	4 (3.1%)	5 (3.7%)	3 (2.2%)	6 (4.5%)	8 (6.1%)	26 (3.9%)
Work in healthcare						
Working in healthcare	9 (7.0%)	14 (10.3%)	12 (9.0%)	10 (7.5%)	12 (9.1%)	57 (8.6%)
Not working in healthcare	119 (93.0%)	122 (89.7%)	122 (91.0%)	123 (92.5%)	120 (90.9%)	606 (91.4%)

### Procedure

The study was conducted online on 31 August 2023, via the non‐commercial survey web application SoSci Survey; participants were recruited and paid via Prolific. They received a remuneration of ₤2.50 for completing the 20‐min survey and 20% (randomly selected after data collection was finished) had the opportunity to receive a bonus payment of up to ₤1.95 depending on their own decisions and the decisions of other participants in the delayed prescription task (see above).

The participants first provided informed consent and answered a set of demographic questions. Then, they were introduced to a hypothetical situation in which they visited the doctor for an ear infection, who gave them a delayed antibiotic prescription and told them that they should monitor their symptoms before starting the treatment. Depending on the experimental condition, they also received the decision support leaflet and the other two components. They received further instructions on the delayed prescription task and were exposed to three comprehension questions to ensure that they understood the instructions. Subsequently, participants played the delayed prescription game, where the decision support leaflet was displayed in every round in the four conditions, including the leaflet. Finally, they answered the decisional conflict items and were redirected to Prolific for remuneration.

### Data analysis

We ran a one‐way ANOVA using inappropriate antibiotic use as the dependent variable and decision support tool condition as the independent variable. We conducted subsequent post hoc tests with Tukey‐adjusted *p*‐values to examine differences between individual conditions (*monitoring*, *value* and *combination hypothesis*). Additionally, we ran a set of planned pooled contrasts, where we tested the differences between the control condition versus the four decision support tool conditions (*decision support hypothesis*). Furthermore, we tested the difference between the individual value integration and active monitoring conditions versus the value integration and active monitoring condition to test the *combination hypothesis*. Additionally, we repeated these analyses using decisional conflict and its subscales as the dependent variables. We also conducted exploratory linear regression analyses to examine whether the covariates age, gender, education, healthcare employment and health status were associated with inappropriate antibiotic use and decisional conflict. We report effect sizes for all analyses—either generalised eta squared (*η*_*G*_^2^) or Cohen's *d*—each accompanied by 95% confidence intervals.

## RESULTS

**FIGURE 2:**
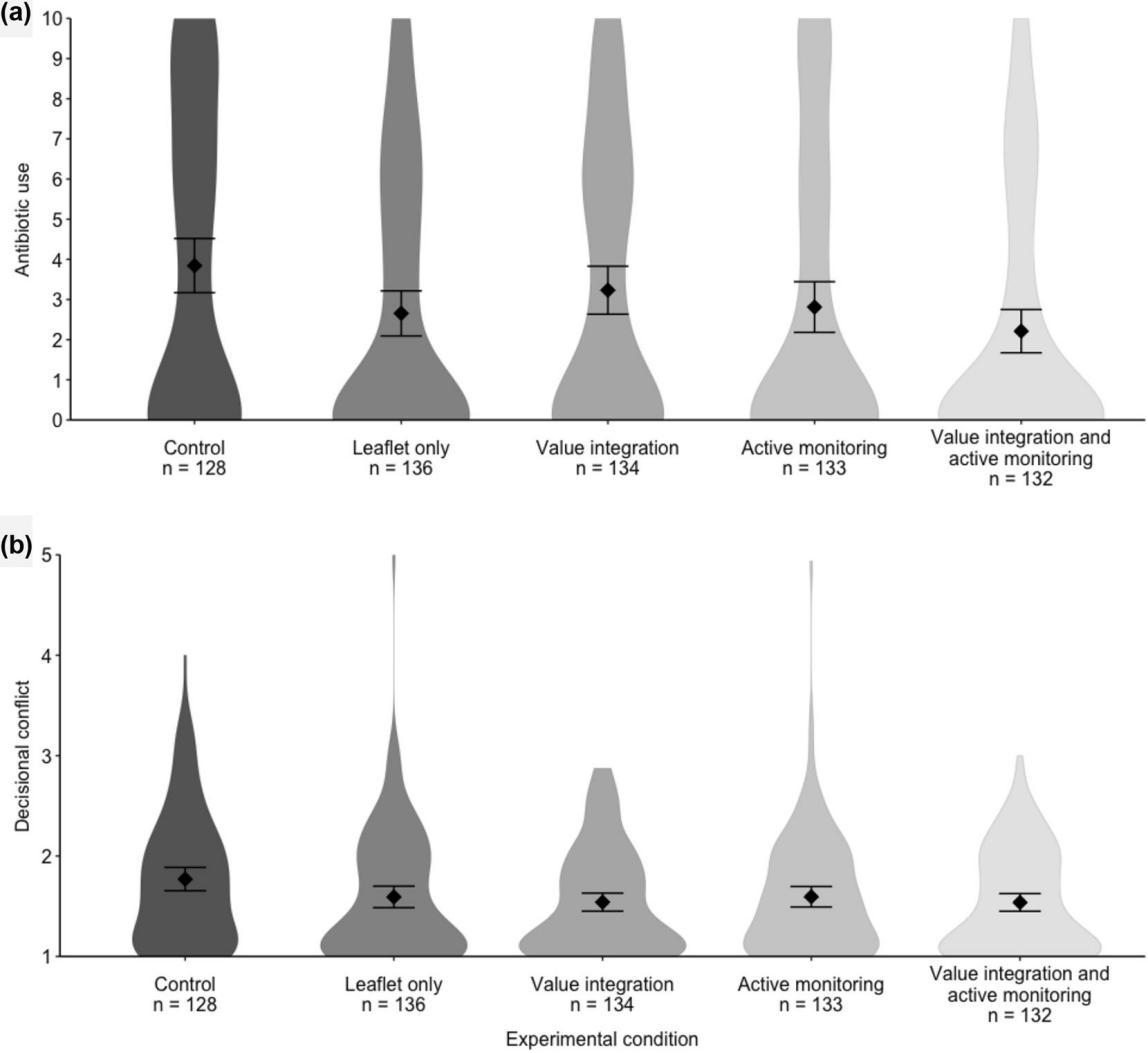
Differences in antibiotic use and decisional conflict across the decision support conditions. *Note*: Results show less antibiotic use (a) and less decisional conflict (b) in the decision support conditions as compared to the control condition. Violin plots show the density distribution, diamonds indicate means and error bars display 95% confidence intervals.

Figure 2a shows that antibiotic use differed significantly by experimental condition, *F*(4, 658) = 49.78, *p* = .003, *η*_*G*_^2^ = .024, 95% CI [0.003, 0.047]. Participants in all leaflet conditions showed less inappropriate antibiotic use than participants in the control condition, *F*(1, 661) = 11.14, *p* < .001, *η*_*G*_^2^ = .016, 95% CI [0.003, 0.041], yielding evidence for the *decision support hypothesis*. Post hoc pairwise comparisons showed that contrary to the *monitoring hypothesis*, participants in the active monitoring condition did not show less inappropriate antibiotic use than participants in the leaflet‐only condition, *t*(267) = 0.30, *p* = .998, *d* = −0.037, 95% CI [−0.276, 0.202]. Likewise, contradicting the *value hypothesis*, participants in the value integration condition did not show less inappropriate antibiotic use than participants in the leaflet‐only condition, *t*(268) = 1.18, *p* = .764, *d* = ‐0.148, 95% CI [−0.386, 0.091]. Participants in the value integration and active monitoring condition showed less inappropriate antibiotic use than the participants in the conditions containing only one of these components, *F*(1, 397) = 4.89, *p* = .028, *η*_*G*_^2^ = .011, 95% CI [0.001, 0.040]. However, these differences were non‐significant when applying Tukey‐adjusted *p*‐values to pairwise comparisons, which is why we conclude that we did not find support for the *combination hypothesis*, *t*(263) = −1.36, *p* = .657, *d* = 0.17, 95% CI [−0.071, 0.411] and *t*(264) = −2.23, *p* = .171, *d* = 0.287, 95% CI [0.046, 0.529], respectively.

### Exploratory analyses

To explore whether demographic factors influenced inappropriate antibiotic use, we conducted a linear regression analysis controlling for age, gender, education, employment in the healthcare sector and self‐reported susceptibility to infections due to a diagnosed health condition. Among these potential covariates, only gender emerged as a significant predictor: Men were more likely than women to misuse antibiotics, *β* = .25, *p* = .001.

Figure 2b shows that decisional conflict differed significantly by experimental condition, *F*(4, 658) = 3.40, *p* = .009, *η*_*G*_^2^ = .021, 95% CI [0.001, 0.041]. Again, we found a treatment effect such that participants in the four leaflet conditions reported significantly less decisional conflict than participants who did not receive the decision support tool, *F*(1, 661) = 12.47, *p* < .001, *η*_*G*_^2^ = .018, 95% CI [0.004, 0.043]. In a linear regression analysis, none of the examined demographic variables (age, gender, education, healthcare employment or health status) were significantly associated with participants' reported decisional conflict (all *p*s > .05).

Regarding the subscales of decisional conflict, we did not find significant differences across the conditions for feeling informed, *F*(4, 658) = 1.82, *p* = .121, *η*_*G*_^2^ = .011, 95% CI [>0.00, 0.027]; experiencing decisional uncertainty, *F*(4, 658) = 1.85, *p* = .118, *η*_*G*_^2^ = .011, 95% CI [>0.000, 0.029]; and feeling like they have made an effective decision, *F*(4, 658) = 2.29, *p* = .058, *η*_*G*_^2^ = .014, 95% CI [>0.000, 0.032]. However, participants differed significantly in clarity regarding their underlying values, *F*(4, 658) = 2.90, *p* = .021, *η*_*G*_^2^ = .017, 95% CI [0.001, 0.036], which was driven again by a treatment effect of the decision support conditions, comparing the control condition to all leaflet conditions: *F*(1, 661) = 7.55, *p* = .006, *η*_*G*_^2^ = .011, 95% CI [>0.000, 0.032]. Participants also differed significantly in how much decisional support they experienced, *F*(4, 658) = 5.48, *p* < .001, *η*_*G*_^2^ = .032, 95% CI [0.008, 0.058]. Likewise, this difference was driven by a treatment effect of the decision support conditions, *F*(1, 661) = 21.60, *p* < .001, *η*_*G*_^2^ = .032, 95% CI [0.010, 0.061].

## DISCUSSION

This study was motivated by the observation that—despite its great potential—the strategy of delayed antibiotic prescribing in primary care remains inefficient (Francis et al., 2012; Llor et al., 2022). Therefore, we investigated whether the use of a patient decision support tool provided as a leaflet that accompanies delayed prescriptions can reduce inappropriate antibiotic use in a hypothetical delayed prescription context.

The results indicate that in all four intervention conditions where participants received the decision support leaflet, they used antibiotics less than participants in the control condition without the leaflet. Thus, given they all had viral infections, their use of antibiotics was less inappropriate. Additionally, participants in the four decision support conditions reported less decisional conflict, particularly in terms of underlying value clarity and experiencing decisional support. This suggests that decision support tools can be helpful during the watchful waiting period of a delayed prescription, as patients can interact with the informational leaflet and engage in some activity, for example, by monitoring their symptoms.

Besides the evaluation of the mainly informational WHO decision support, we investigated whether adding two psychological interventions (a symptom diary and value elicitation) improved its effectiveness. Although we did not find additional benefits (nor evidence of a negative effect), our findings nonetheless offer theoretical insights into why the simple intervention of a decision support leaflet can effectively reduce inappropriate antibiotic use in delayed prescribing contexts. Explorative analyses also indicated that men were more likely than women to misuse antibiotics, in line with meta‐analytic evidence that men engage in more health risk‐taking behaviour than women (Byrnes et al., 1999) and that men are more likely than women to adopt pharmaceutical health‐protective behaviours such as medication use (Moran & Del Valle, 2016).

In line with the COM‐B model (Michie et al., 2011), the leaflet has strengthened key behavioural drivers through enhancing psychological capability by making it clear when and how to act, offering a structural way to interact with the recommendation to wait and strengthening reflective motivation by outlining the rationale for delayed prescribing. The observed decrease in decisional conflict further suggests an increase in psychological capability and reflective motivation by feeling more supported and less uncertain during the decision‐making process. Although we tested leaflet conditions with additional psychological components, namely, active monitoring and value integration, these modifications did not significantly outperform the standard version. From a self‐determination theory (Ryan & Deci, 2000) perspective, this suggests that the standard leaflet may already provide sufficient support for informed, autonomous decision‐making. The lack of additional benefit from the extended versions may also reflect increased cognitive load or perceived redundancy. Simplifying decision support tools by focusing on essential components may enhance usability without compromising efficacy. Although we captured decision‐related uncertainty via decisional conflict, we did not include a direct measure of infection certainty, limiting the ability to test the proposed mechanism that the active monitoring component influences perceived infection certainty.

These findings also have implications for clinical practice. In typical consultations, time constraints and diagnostic uncertainty make it difficult for general practitioners to provide detailed follow‐up guidance (O'Riordan et al., 2011). When it comes to delayed prescriptions, this is especially challenging because patients are expected to self‐monitor and make treatment decisions after the clinical consultation, often without further clinical input. Our results suggest that a simple, low‐cost decision support leaflet can reduce inappropriate antibiotic use without requiring major changes to clinical workflows, although in our study the leaflet was presented repeatedly, and may thus represent a relatively high dose of exposure compared with usual care. The decision support leaflet can bridge the gap between initial clinical advice and subsequent self‐management, particularly in moments when patients experience uncertainty or pressure to ‘do something’. As such, this tool represents a scalable way to strengthen antibiotic stewardship in primary care.

Current decision support tools for antibiotic use are either aimed at healthcare professionals (Ashiru‐Oredope et al., 2020; McIsaac et al., 2017; Steurer et al., 2011) or are used to promote shared decision‐making during the doctor's consultation (Coronado‐Vázquez et al., 2020). The present study is important because it demonstrates the usefulness of a decision support tool targeted at patients during the delayed prescription period.

### Limitations

This study has some limitations. First, the delayed prescription task is a hypothetical decision task within a simplified decision environment. Despite the many benefits and strengths of this method (Böhm et al., 2016, 2022; Chapman et al., 2012; Korn et al., 2020; Santana et al., 2023, 2024), it lacks the complexity of medical decision‐making in real life, where symptoms are experienced bodily, frequently overlap across illnesses and uncertainty complicates treatment decisions. While the use of behaviour‐contingent monetary incentives has been validated in prior work (Böhm et al., 2016, 2022; Chapman et al., 2012; Korn et al., 2020; Santana et al., 2023, 2024), it cannot fully capture the emotional and physical aspects of medical decision‐making. However, these tasks provide a low‐cost, scalable way to separate intervention effects and explore underlying mechanisms in a controlled environment. Future research should extend these findings by combining experimental studies with clinical trials to validate the behavioural insights and assess the impact of decision support tools under actual conditions.

Second, it is important to note that the decision support leaflet is intended as a participatory shared decision‐making tool that general practitioners and patients discuss together. This interactive element was not evaluated here. We only evaluated the mere informational component of the WHO decision support tool leaflet. Therefore, the effects we observed should be regarded as a conservative estimate and may even be larger when exploiting the decision support's interactive components in practice.

Lastly, the study focuses on the context of delayed prescribing in a specific disease context (i.e. ear infection) and a specific study population (i.e. UK adults recruited through Prolific). The sample overrepresents English‐speaking, highly educated individuals, with over 60% holding a bachelor's degree or higher, and all participants having internet access. Additional sociodemographic factors known to influence health behaviours, such as race, income, urbanicity and health literacy were not collected. Research has shown that higher deprivation levels are associated with increased antibiotic prescribing (Thomson et al., 2020) and that individuals from minority ethnic groups differ in their awareness and attitudes towards antibiotic use (Schuts et al., 2019). Geographic differences, such as in urban versus rural areas, further impact antibiotic prescribing patterns (Curtis et al., 2019). Moreover, individuals with limited health literacy are more likely to misuse antibiotics and less likely to accurately understand antibiotic purposes and risks (Elkhadry & Tahoon, 2024; Muflih et al., 2021).

Future research should aim to recruit more representative samples and consider how sociodemographic factors influence the effectiveness of decision support tools. Hence, the main finding (that a decision support tool is useful in reducing inappropriate antibiotic use in cases of diagnostic uncertainty) is not automatically generalisable to other contexts, such as populations with different cultural values, socio‐economic backgrounds and other regulations regarding antibiotics (e.g. over‐the‐counter sale of antibiotics).

### Conclusion

While delayed antibiotic prescribing has shown promise for reducing inappropriate antibiotic use, its effectiveness falls short of its full potential because many patients continue to use antibiotics inappropriately. Our study provides causal evidence—within a controlled, hypothetical decision setting—that decision support tools, such as the WHO's leaflet evaluated in this study, can promote more appropriate antibiotic use by providing patients with information and tools that are relevant to the delayed prescription period, during which patients experience uncertainty and feel the need to take action.

## CONFLICT OF INTEREST STATEMENT

The authors have no conflicts of interest to declare.

## ETHICS STATEMENT

Ethical approval was granted by the University of Vienna (2023/W/015A).

## Supporting information


**Figure S1.** Adapted WHO Decision Aid.
**Figure S2.** Value Elicitation Task.
**Figure S3.** Symptom Monitoring Task.

## Data Availability

The data that support the findings of this study are openly available in Open Science Framework at https://osf.io/5hscn.
