# Supplementary material for: Using a decision support tool to reduce inappropriate antibiotic use after a delayed prescription
Source: Appl Psychol Health Well Being. 2026 Aug 7;18(4):e70198. doi: 10.1111/aphw.70198 (PMC13451548; doi:10.1111/aphw.70198)
Supplement: Supplementary file 1 [file APHW-18-0-s001.docx]

**Online Supplement**

**Using a decision support tool to reduce inappropriate antibiotic use after a delayed prescription**

Adapted Decision Aid	Page 2

Symptom Monitoring Task	Page 4

Value Elicitation Task	Page 5

**Figure S1**

*Adapted WHO Decision Aid*

(Original version is available via: https://who.canto.global/v/UFPS6IVVV4/allfiles?viewIndex=0&auth=sso)

**Figure S2**

*Value Elicitation Task*

**Figure S3**

*Symptom Monitoring Task*
